# Added survival benefit of whole brain radiotherapy in brain metastatic non-small cell lung cancer: Development and external validation of an individual prediction model

**DOI:** 10.3389/fonc.2022.911835

**Published:** 2022-11-29

**Authors:** Kyrhatii Trikhirhisthit, Jiraporn Setakornnukul, Kullathorn Thephamongkhol

**Affiliations:** ^1^ Division of Radiation Oncology, Department of Radiology, Faculty of Medicine, Siriraj Hospital, Mahidol University, Bangkok, Thailand; ^2^ Division of Radiation Oncology, Department of Radiology, Sawanpracharak Hospital, Nakhonsawan, Thailand

**Keywords:** brain metastases, individual prediction model, non-small cell lung cancer, whole brain radiotherapy, overall survival

## Abstract

**Background:**

The heterogeneous survival benefit of whole brain radiotherapy (WBRT) in brain metastatic non-small cell lung cancer (NSCLC) was prospectively evidenced in the Quality of Life after Treatment for Brain Metastases (QUARTZ) trial, resulting in inconsistent guideline recommendations and diverse clinical practices for giving WBRT. The objective of this study was to develop and externally validate an individual prediction model to demonstrate the added survival benefit of WBRT to assist decision making when giving WBRT is undetermined.

**Methods:**

For model development, we collected 479 brain metastatic NSCLC patients unfit for surgery or stereotactic radiotherapy techniques at Siriraj Hospital. Potential predictors were age, sex, performance status, histology, genetic mutation, neurological symptoms, extracranial disease, previous systemic treatment, measurable lesions, further systemic treatment, and WBRT. Cox proportional hazard regression was used for survival analysis. We used multiple imputations to handle missing data and a backward selection method for predictor selection. Bootstrapping was used for internal validation, while model performance was assessed with discrimination (c-index) and calibration prediction accuracy. The final model was transformed into a nomogram and a web-based calculator. An independent cohort from Sawanpracharak Hospital was used for external validation.

**Results:**

In total, 452 patients in the development cohort died. The median survival time was 4.4 (95% CI, 3.8–4.9) months, with 5.1 months for patients who received WBRT and 2.3 months for those treated with optimal supportive care (OSC). The final model contained favorable predictors: female sex, KPS > 70, receiving additional systemic treatment, and WBRT. Having active extracranial disease, experiencing neurological symptoms, and receiving previous systemic treatment were adverse predictors. After optimism correction, the apparent c-index dropped from 0.71 (95% CI, 0.69–0.74) to 0.70 (95% CI, 0.69–0.73). The predicted and observed values agreed well in all risk groups. Our model performed well in the external validation cohort, with a c-index of 0.66 (95% CI, 0.59–0.73) and an acceptable calibration.

**Conclusions:**

This model (https://siriraj-brainmetscore.netlify.app/) predicted the added survival benefit of WBRT for individual brain metastatic NSCLC patients, with satisfactory performance in the development and validation cohorts. The results certify its value in aiding treatment decision-making when the administration of WBRT is unclear.

## Introduction

The standard role of palliative WBRT was recently challenged in the QUARTZ trial (1). In this noninferiority study, patients with brain metastases from primary NSCLC who were inoperable or unsuitable for stereotactic radiotherapy were randomly assigned to receive WBRT or OSC, including dexamethasone. There was no difference in the survival rates of the 2 treatment groups. However, planned subgroup analyses showed heterogeneity in survival in favor of WBRT for patients younger than 60 years. The analyses also revealed potential survival benefits with WBRT in patients with a good Karnofsky performance status (KPS score ≥ 70%), no extracranial metastases, and controlled primary NSCLC.

Two international guidelines inconsistently recommend the omission of WBRT. The British National Institute for Health and Care Excellence does not recommend WBRT for NSCLC patients with brain metastases that are not suitable for surgery or stereotactic techniques and whose KPS score is < 70% (2). The US National Comprehensive Cancer Network suggests that it is reasonable to delay radiation use for patients with small asymptomatic brain metastases with actionable mutations if there is an active agent with brain penetration (3). In our practice, treating physicians also consider the status of primary lung and other extracranial diseases, actionable mutations, and the availability of systemic treatment, including targeted therapy. This approach results in diverse clinician preferences and debate as to which patients should be selected for WBRT.

Several survival prediction models for brain metastases have been developed to guide clinical decisions (4–16) (**Table S1**). However, neither WBRT nor systemic treatment (targeted therapy) has been used as a prognostic factor in the models currently available. Thus, to evaluate the added survival benefits of WBRT, the development and validation of an individual prediction model using WBRT as a main prognostic factor is the objective of this study.

## Methods

### Study design and participants

We conducted a retrospective cohort study. Patients were eligible if they were 18 years or older and had histologically proven primary NSCLC with brain metastases that had been confirmed by computed tomography or magnetic resonance imaging. Patients were excluded if they received tumor removal, stereotactic radiosurgery (SRS), or stereotactic radiotherapy (SRT). A Siriraj Institutional Review Board-approved database was generated for model development. It was made up of NSCLC patients with brain metastases (N = 479) treated with WBRT or OSC. They had been treated at Siriraj Hospital, a teaching medical center in Thailand, between January 2004 and December 2019. For the independent validation cohort, we collected data related to 100 NSCLC patients with brain metastases treated between January 2017 and June 2018 at Sawanpracharak Hospital, a tertiary care center in northern Thailand.

As neither center had a formal policy of selecting suitable patients for palliative systemic treatment or WBRT, decisions were made by individual oncologists. During the study period, as radiation oncologists, the following were general considerations for treatment options: 1) Patients with mass effect underwent surgery 2) SRS was administered to patients with fewer than four brain metastasis lesions and no extracranial progression within the previous three months. 3) patients with at least four brain metastasis lesions received WBRT. During the study period, WBRT was administered without avoiding the hippocampal region. The WBRT regimens used at the 2 medical centers were similar, with 30 Gy in 10 fractions or 20 Gy in 5 fractions commonly administered. The systemic treatment options at Siriraj Hospital were chemotherapy, targeted therapy, and immunotherapy, while chemotherapy was the only option available at Sawanpracharak Hospital.

Data were collected and managed using Research Electronic Data Capture (REDCap) electronic data capture tools hosted at Siriraj Centre of Excellence in Bioinformatics and Data Management, Faculty of Medicine Siriraj Hospital, Mahidol University. REDCap is a web-based, secure application designed to support data capture for research studies. It provides (1) an intuitive interface for validated data entry; (2) audit trails to track data manipulation and export; (3) automated export procedures for seamless data downloads to standard statistical packages; and (4) procedures to import data from external sources (17).

### Predictors and outcome variables

The primary outcome was overall survival, calculated from the diagnosis of brain metastasis to the date of death from any cause or to the last follow-up. The final statuses of the patients were determined as at November 30, 2020, using local death registry data and hospital records. Patients who survived until this date were censored for the computation of overall survival.

Previously established prognostic factors for survival in brain metastatic NSCLC patients were collected as potential predictors, while WBRT was a mandatory predictor in our model. We also proposed new potential predictors related to systemic treatment (**Table S2**). The predictor and outcome evaluations were identical for the 2 data sets.

### Statistical analysis

The analyses and reports followed the guidelines of TRIPOD (Transparent Reporting of a Multivariable Prediction Model for Individual Prognosis or Diagnosis) (18). Of the 479 patients accrued in the development cohort, 452 had died by the time of analysis. The rule of thumb of 10 outcome events per variable was adopted (19, 20). At least 45 parameters were adequately examined in our model. We assumed that 100 events would occur in the validation cohort and would be sufficient for external validation. Cox proportional hazard regression was used for survival analyses. Proportional hazard assumptions were tested using log-log plots and Schoenfeld residuals. A linearity test for continuous variables was performed using Martingale residuals (21). Almost 30% of the data were unavailable for complete case analysis. To handle missing data, we used multiple imputations with chained equations (22, 23). Thirty imputations were performed on the complete data set of all participants using identical known information. Multiple imputations were performed using mi impute chained and mi estimate commands. A backward elimination method was used to decide which potential predictors should be included in our reduced model based on the Akaike information criterion, keeping predictors with a P value of less than 0.157 (24). Again, WBRT was our mandatory predictor.

Model performance was evaluated through 2 fundamental aspects: discrimination and calibration (25–27). Discrimination in our situation is the model’s ability to predict which patients with brain metastases will die earlier and which will die later or not at all, estimated by the concordance index (c-index). A c-index close to 1.0 indicates excellent discrimination, whereas 0·5 indicates no discrimination beyond chance. Calibration measures how well the predicted absolute risk of death corresponds to the actual (observed) risk of mortality. In our study, calibration is first reported graphically using a calibration plot, in which the predicted risk is plotted against the observed incidence of the outcome in 10 risk groups. These groups were generated by linear predictors, and the outcomes were split into 10 equal deciles. Perfect calibration shows prediction on the 45-degree line of the calibration plot. Calibration is also reported through plots of the predicted and observed survival curves for 3 risk groups (low, intermediate, and high), based on the linear predictor distribution using the 25th and 75th centile cutoffs to assess longitudinal calibration. The apparent performance of a fitted model can be inflated due to overfitting (28). Therefore, to evaluate the potential for overfitting of our developed models, we performed bootstrapping (27, 29), a random resampling with replacement using the rms package for internal validation. After 200 samples, we combined the estimates across imputed data sets using Rubin’s rules (30, 31) to generate an optimism-corrected c-index and calibration slope. To derive the risk score of the final model, we adjusted the coefficients of the reduced model for optimism using the calibration slope as a shrinkage factor. The baseline survival probabilities (S0) are presented for 3 (S0[3]) and 6 (S0[6]) months. The probability of survival at specific time points was predicted using (S0) exp (β1x1+… + βnxn), where β1–βn are the coefficients for each predictor and x1-xn are the predictor values. The sum of βx represents individual risk scores. The final model was transformed into a nomogram using the nomogram function of the rms package. Finally, we generated a web-based calculator for individual survival prediction.

To externally validate our newly developed prediction model, we used a separate data set from Sawanpracharak Hospital. The predictive performance of our final model using this independent data set was also evaluated in terms of discrimination and calibration (32). Finally, the performance of our model was compared to the widely used Graded Prognostic Assessment for Lung Cancer Using Molecular Markers (Lung-molGPA) index (15). Using the coefficients in the Lung-molGPA index (**Table S3**), we generated linear predictors to estimate the c-index for performance comparison. Analyses were conducted with Stata/SE, release 14 (StataCorp LP, College Station, TX, USA) and R, version 3.9 (R Foundation for Statistical Computing, Vienna, Austria).

## Results

### Participants and missing values

**Figure 1:**
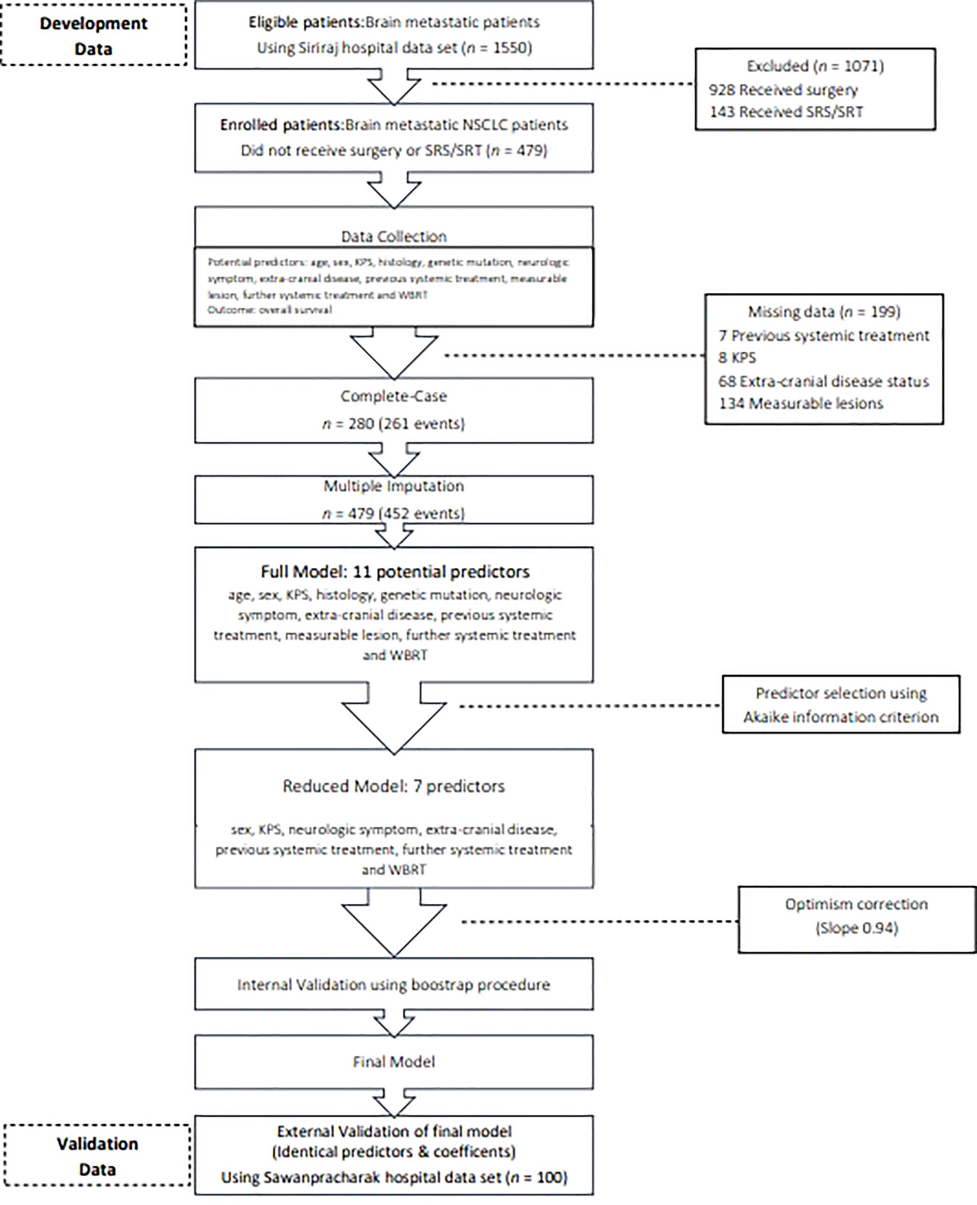
Flow diagram. KPS, Karnofsky performance status; NSCLC. non-small cell lung cancer; SRS, stereotactic radiosurgery; SRT, stereotactic radiotherapy; WBRT, whole brain radiotherapy.

The characteristics of the participants in the development and validation cohorts are listed in **Table 1**. Most of the patients had adenocarcinoma with brain metastasis. In the development cohort, 389 patients received WBRT, and 90 patients received OSC. Patients who received WBRT had better KPS scores (65.5% of the WBRT subgroup had scores ≥ 70%, compared with only 38.5% of the OSC subgroup). Furthermore, more patients who received WBRT presented with milder symptoms (39.8% vs. 25.3%), and they were more likely to receive additional systemic treatment (35.7% vs. 15.2%). Patients receiving WBRT also had better prognoses, according to the RPA and LungMolGPA indices. Fourty seven and twenty-five percent of patients had at least 4 lesions in WBRT and OSC group, respectively. The mean largest diameters were 3.1 cm and 3.3 cm in OSC group and WBRT group. Genetic mutations were not tested in three-quarters of the development cohort. Two hundred eighty records were found in the complete case data set, contributing to 261 events (**Figure 1**). The status of extracranial disease and the presence of measurable lesions were critical missing values. Regarding multiple imputations, 452 events were obtained (364 who received WBRT and 88 who were treated with OSC). The median survival times were 5.1 and 2.3 months for the WBRT and OSC groups, respectively (**Figure S1**). The median follow-up time was 4.3 (95% CI, 1.0–8.4) months.

**Table 1:** Clinical characteristics.

	DEVELOPMENT			EXTERNAL VALIDATION		
Characteristics	WBRT	OSC	Whole cohort	WBRT	OSC	Whole cohort
	*n* = 389(81.2%)	*n* = 90(18.8%)	*n* = 479	*n* = 76(76%)	*n* = 24(24%)	*n* = 100
Patient profile						
Age (mean ± SD)	60.6 ± 11.2	63.1 ± 12.3	61.1 ± 11.4	63.2 ± 1.0	61.8 ± 10.3	62.8 ± 9.2
Female	162 (41.7%)	47 (51.1%)	209 (43.5%)	31 (41%)	12 (50%)	43 (43.0%)
KPS ≥ 70%	250 (65.5%)	35 (38.5%)	285 (60.3%)	48 (63%)	6 (25%)	54 (54.0%)
Disease profile						
Histology						
- Adenocarcinoma	304 (78.2%)	76 (84.4%)	380 (79.3%)	58 (76%)	18 (75%)	76 (76.0%)
- Non-adenocarcinoma	85 (21.8%)	14 (15.6%)	99 (20.7%)	18 (24%)	6 (25%)	24 (24.0%)
EGFR/ALK mutation						
- Positive	44 (11.3%)	15 (16.3%)	62 (13.0%)	7 (9%)	3 (13%)	10 (10.0%)
- Negative	47 (12.1%)	11 (12.0%)	55 (11.4%)	6 (8%)	1 (4%)	7 (7.0%)
- Unknown	298 (76.6%)	66 (71.7%)	364 (75.7%)	63 (83%)	20 (83%)	83 (83.0%)
Neurological symptoms						
- None	48 (12.3%)	8 (8.8%)	56 (11.7%)	3 (4%)	0 (0%)	3 (3.0%)
- Mild	107 (27.5%)	15 (16.5%)	122 (25.4%)	13 (17%)	1 (4%)	14 (14.0%)
- Major	234 (60.2%)	68 (74.7%)	302 (62.9%)	59 (79%)	23 (96%)	82 (83.0%)
Extra-cranial disease						
- Controlled lung and no ECM	28 (8.4%)	5 (6.4%)	33 (8.0%)	5 (7%)	0 (0%)	5 (5.0%)
- Controlled lung & ECM	43 (12.8%)	6 (7.7%)	49 (11.9%)	17 (23%)	6 (29%)	23 (24.0%)
- Uncontrolled lung or ECM	58 (17.3%)	15 (19.2%)	73 (17.7%)	7 (9%)	2 (10%)	9 (9.0%)
- First diagnosis lung with any ECM	206 (61.5%)	52 (66.7%)	258 (62.5%)	45 (61%)	13 (62%)	58 (61.0%)
Previous systemic treatment	159 (41.6%)	35 (38.0%)	194 (40.9%)	12 (50%)	40 (40.0%)
Further systemic treatment	139 (35.7%)	14 (15.2%)	153 (31.8%)	15 (21%)	0 (0%)	15 (15.0%)
RPA						
- Class I	33 (9.5%)	2 (2.4%)	35 (8.1%)	3 (4.0%)	1 (4.4%)	4 (4.08%)
- Class II	197 (56.5%)	31 (37.4%)	228 (52.8%)	46 (61.3%)	6 (26.1%)	52 (53.1%)
- Class III	119 (34.1%)	50 (60.2%)	169 (39.1%)	42 (42.7%)	16 (69.6%)	42 (42.9%)
LungMol GPA						
▪ Adenocarcinoma						
- GPA 0–1	105 (39.0%)	29 (56.9%)	134 (41.9%)	20 (46.5%)	11 (78.6%)	31 (54.4%)
- GPA 1.5–2	115 (42.8%)	15 (29.4%)	130 (40.6%)	16 (37.2%)	2 (14.3%)	18 (31.6%)
- GPA 2.5–3	45 (16.7%)	7 (13.7%)	52 (16.3%)	7 (16.2%)	1 (7.1%)	8 (14%)
- GPA 3.5–4	4 (1.5%)	–	4 (1.3%)	–	–	–
▪ Non-adenocarcinoma						
- GPA 0–1	25 (33.8%)	6 (50%)	31 (36.1%)	2 (12.5%)	4 (100%)	6 (30%)
- GPA 1.5–2	36 (48.7%)	5 (41.7%)	41 (47.7%)	10 (62.5%)	–	10 (50%)
- GPA 2.5–3	13 (17.6%)	1 (8.3%)	14 (16.3%)	4 (25%)	–	4 (20%)

ALK, anaplastic lymphoma kinase gene; ECM, extracranial metastasis; EGFR, epidermal growth factor receptor; KPS, Karnofsky performance status; M, missing values; OSC, optimal supportive care; WBRT, whole brain radiotherapy.

### Model development

**Table S4** details the results of univariable and multivariable Cox regression analyses for overall survival. Age was the only continuous variable and was found to have a good linear relationship with survival. To avoid information loss, we did not perform a categorization (18). The proportional hazard assumption was satisfied. In the univariable analyses, a KPS score > 70%, epidermal growth factor receptor/anaplastic lymphoma kinase gene (EGFR/ALK) mutation, receiving further systemic treatment, and WBRT were significantly associated with a reduced risk of death. Conversely, major neurological symptoms, uncontrolled extracranial disease, and newly diagnosed lung cancer were significantly associated with death. In the multivariable model, the EGFR/ALK mutation, extracranial disease status, receipt of additional systemic treatment, and WBRT remained significant variables. After dropping the candidate predictors listed in **Table S2** stepwise based on the Akaike information criterion, the following variables were included in the reduced model: sex, KPS, neurological symptoms, extracranial disease, previous systemic treatment, further systemic treatment, and WBRT (**Table 2**). WBRT remained significant and was retained in the reduced model without being forced back. It should be noted that WBRT exhibited a negative coefficient, indicating that it is a good predictor.

**Table 2:** Comparison of model’s coefficients.

Characteristics	Complete case	*P* value	Imputation (full model)	*P* value	Reduced model^a^	*P* value	Final Model^b^
Age	0.008	0.137	-0.001	0.834	–	–	–
Female	-0.123	0.346	-0.155	0.122	-0.17	0.086	-0.16
KPS score > 70%	-0.242	0.089	-0.341	0.002	-0.334	0.002	-0.317
Histology							
- Adenocarcinoma	–	–	–	–	–	–	–
- Nonadenocarcinoma	-0.029	0.852	-0.013	0.913			
EGFR/ALK mutation							
- Negative	–	–	–	–	–	–	–
- Positive	-0.623	0.025	-0.155	0.474			
- Unknown	0.072	0.725	0.054	0.733			
Neurological symptoms							
- None	–	–	–	–	–	–	–
- Mild	0.054	0.814	0.158	0.386	0.17	0.346	0.16
- Major	0.321	0.135	0.311	0.073	0.324	0.052	0.305
Measurable lesions							
- Absence	–	–	–	–	–	–	–
- Presence	-0.071	0.681	-0.016	0.928			
Extracranial disease							
- Controlled lung and no ECM	–	–	–	–	–	–	–
- Controlled lung & ECM	0.934	0.001	0.599	0.011	0.601	0.009	0.565
- Uncontrolled lung or ECM	1.064	< 0.001	0.857	< 0.001	0.868	< 0.001	0.816
- First Dx lung with any ECM	1.387	< 0.001	1.028	< 0.001	1.038	< 0.001	0.977
Previous systemic treatment	0.374	0.051	0.294	0.079	0.283	0.078	0.271
Received further systemic treatment	-0.926	< 0.001	-0.973	< 0.001	-1.022	< 0.001	-0.961
WBRT	-0.693	< 0.001	-0.529	< 0.001	-0.514	< 0.001	-0.483

ECM, extra-cranial metastasis; N/A, not applicable; WBRT, whole brain radiotherapy ^a^ Reduced model from imputation set before optimism correction; ^b^ Final model after optimism correction using slope of 0.94.

### Apparent performance and internal validation

**Figure 2:**
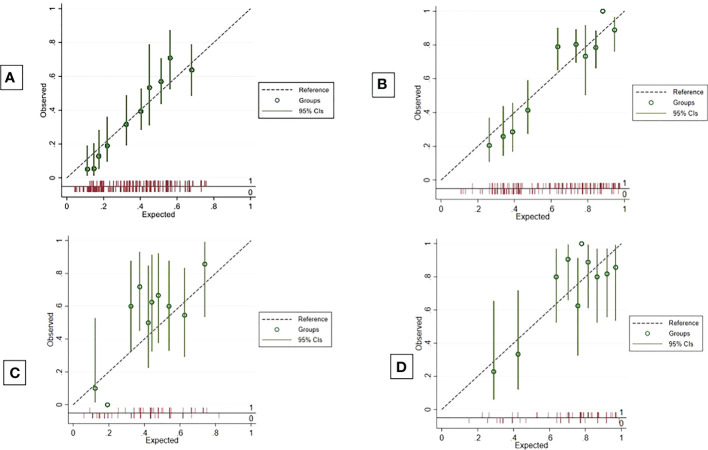
Calibration plots. Calibration plots to predict 3-month and 6-month overall survival probabilities in development cohort **(A, B)** and validation cohort **(C, D)**.

**Figure 3:**
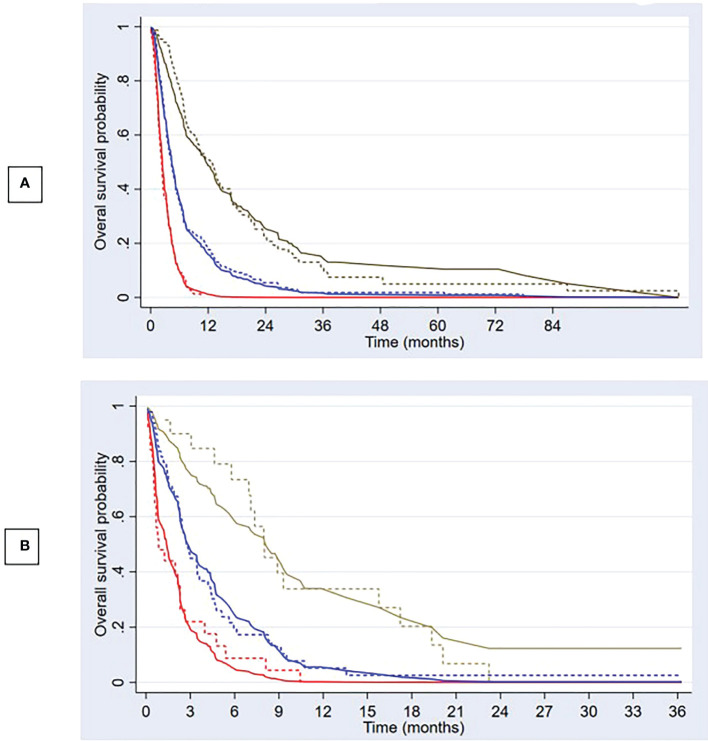
Predicted overall survival and observed Kaplan–Meier curves Predicted overall survival (solid lines) compared with observed Kaplan–Meier curves (dashed lines) in development **(A)** and validation cohort **(B)** for 3 risk groups; low risk (brown), intermediate risk (blue), and high risk (red).

The apparent c-index was 0.71 (95% CI, 0.69–0.74) in the reduced model. Calibration plots for 3- and 6-month overall survival appeared to be well-calibrated (**Figures 2A, B**). **Figure 3A** also illustrates that the predicted and observed risks agreed well in all risk groups. Internal validation using bootstrapping provided a corrected c-index of 0.70 (95% CI, 0.69–0.73) and a calibration slope of 0.94 for coefficient adjustment. The final model coefficients after optimism correction are presented in **Table 2**. They are available for comparison among the developed models. Survival probabilities can be predicted using the equation shown in **Supplementary Table S5**.

### Model presentation and application

The final score-transformed nomogram is shown in **Supplementary Figure S2**. The median survival time can be individually predicted by summation of the predictor values, and it is best visualized in **Supplementary Figure S3**. We found that the higher the calculated points were, the shorter the survival time was. The web-based model to predict median survival time and the survival probabilities at specific time points are available online at https://siriraj-brainmetscore.netlify.app/. The added survival benefit of WBRT is also displayed on the website. The most significant benefits of WBRT were found in patients who had controlled lung disease without extracranial metastases and in patients who received further systemic treatment.

### External validation and model comparison

Compared to the development cohort, the validation cohort showed a similar distribution of characteristics for the 2 treatment groups (**Table 1**). However, the patients in the validation set had worse neurological symptoms and were less likely to receive further systemic treatment than the patients in the development cohort. Less than 20% of EGFR/ALK mutations were tested in the validation cohort. The c-index of the validation data set dropped to 0.66 (95% CI, 0.59–0.73). The model slightly overestimated the survival probabilities of the low- and high-risk groups. However, minimal underestimation was observed for the intermediate-risk group (**Figure 3B**). The calibration plots for overall survival at 3^rd^ and 6^th^ month showed negligible miscalibration (**Figures 2C, D**). Compared to the performance of our model, the Lung-molGPA index performed poorer in our data set, with c-indices of 0.61 (95% CI, 0.58–0.65) and 0.58 (95% CI, 0.51–0.65) for the histology of adenocarcinoma and non-adenocarcinoma, respectively.

### Additional analysis

Modest information on the status of genetic mutations was examined in both data sets, which raised our concern. Therefore, we performed a separate analysis on 117 patients with known genetic mutation profiles, in which 73 observations and 61 events were found in the complete case analysis. Using the identical model-building technique, the final model in this subgroup analysis included only 3 predictors: extracranial disease, receiving further systemic treatment, and WBRT. Again, WBRT was significant and remained in the model without needing to be forced back. The genetic profile variable was removed from the model in the same way as we did with the original cohort, resulting in a negligible decline in the c-index from 0.75 to 0.73. Further analysis using lesion number and size as candidate predictors was also performed. These two predictors were dropped out from the final model.

## Discussion

We have developed and externally validated an individual survival prediction model for brain metastatic NSCLC patients who have not received surgery or SRS/SRT. This model contains several favorable predictors: female sex, a KPS score of > 70, receiving further systemic treatment, and WBRT. The adverse predictors in the model are active extracranial disease, experiencing neurological symptoms, and receiving previous systemic treatment. The model was developed in 2 different forms for ease of use: a nomogram and a web-based calculator. The added survival benefit of WBRT can be clearly and individually appreciated with the web-based version. Two characteristics that demonstrate the most significant benefits of WBRT are (1) patients who have controlled primary lung cancer without extracranial metastases and (2) patients who have received further systemic treatment.

Our model was derived following the recommendations of the TRIPOD statement (18). We comprehensively considered all methodological aspects, including transparency of data sources, the adequacy of events per predictor, missing data imputations, and the unambiguous model-building process. Our selected predictors are used routinely in clinical practice and have explicit definitions and coding. We also simplified the complicated mathematical equation into a straightforward input-output web use. This user-friendly interface would facilitate effective communication with patients about the risks and benefits of WBRT. A prediction model should not enter clinical practice without proven and value-adding performance (33). It is also crucial for a model to maintain its ability in an independent data set, in other words, to be externally validated. Our model demonstrated successful predictive performance for discrimination and calibration in the original cohort, with slightly poorer but still acceptable performance in the separate data set. These results assure its generalizability.

Numerous survival prediction models have been developed for patients with metastatic brain NSCLC (6, 7, 13–16). The well-known Lung-molGPA index constructed by Sperduto et al. (15) was updated in 2016 by incorporating a new predictor, genetic status. Participants involved in the development of the Lung-molGPA index received WBRT, surgery, stereotactic radiosurgery, or a combination of these treatments. The overall median survival time in the Lung-molGPA cohort (12 months) was longer than in our study and the QUARTZ trial. The authors did not report the predictive performance of this widely used index. In the present study, we validated the Lung-molGPA index using our database and found an inferior discriminative performance compared to our model. Unfortunately, the prediction accuracy, also known as calibration of the Lung-molGPA index, remained doubtful. The recent nomogram generated by Agarwal et al. (16) at Tata Memorial Hospital in India aimed to identify patients who may not benefit from WBRT by predicting 70-day and 140-day survival probabilities. Participants in the Indian cohort had a poor prognosis, as in the QUARTZ trial, with overall median survival of 5.5 months. This result was comparable with our study. However, the nomogram developed by Agarwal and colleagues was only internally validated, and it had a lower C-index of 0.64, with a moderate degree of calibration error. In contrast, our prediction model, which has been developed and externally evaluated, demonstrates superior discrimination (0.71 in developed data and 0.66 in validated data) and good calibration. In addition, we like to emphasize the need of introducing WBRT as a new critical prognostic factor for patients with a poor prognosis, as in the QUART trial, as well as for refractory patients in western countries who have no systemic therapy options available.

This current work has 2 limitations. First, our participants represented only brain metastatic NSCLC patients who had a poor prognosis and were unsuitable for surgery or SRS/SRT. The reproducibility of the model for patients with good prognoses should be used with caution, and we suggest that clinicians use our model whenever the role of WBRT is in doubt. Second, our findings originated in a country with limited access to molecular testing, third-generation EGFR TKIs, and second-generation ALK inhibitors. In addition, the genetic mutation status, a well-known predictor, was excluded from our statistical model. However, our additional analysis found that the remaining predictors provided adequate information for discriminatory performance.

In conclusion, our model demonstrated the added survival benefit of WBRT for individual patients with satisfactory performance in terms of discrimination and calibration for both the development and validation cohorts. The web-based model to predict median survival time and the survival probabilities at specific time points are available online at https://siriraj-brainmetscore.netlify.app/. This tool can be used to help informs as to why the patient may or may not be offering WBRT. The findings confirm its beneficial role for vulnerable patients with metastatic brain NSCLC when the administration of WBRT is unclear.

## Data availability statement

The raw data supporting the conclusions of this article will be made available by the authors, without undue reservation.

## Ethics statement

The studies involving human participants were reviewed and approved by Siriraj Institutional Review Board and Ethics Committee in Human Research Sawanpracharak Hospital. Written informed consent for participation was not required for this study in accordance with the national legislation and the institutional requirements.

## Author contributions

KyT and KuT had full access to all data in the study and take responsibility for the integrity of the data and the accuracy of the data analysis. Original research concept: JS. Modifying concept to unique research question, feasible data collection and methodological design: all authors. Acquisition, analysis, or interpretation of data: all authors. Drafting of the manuscript: KyT. Critical revision of the manuscript for important intellectual content: KuT and JS. Statistical analysis: KyT and KuT. Obtained funding: KuT. All authors contributed to the article and approved the submitted version.

## Funding

This study was funded by the Siriraj Reseach Fund Faculty of Medicine, Siriraj Hospital, Mahidol University, grant number R016331010 and Sawanpracharak Hospital Medical Education Center, grant number 05/2565.

## Conflict of interest

The authors declare that the research was conducted in the absence of any commercial or financial relationships that could be construed as a potential conflict of interest.

## Publisher’s note

All claims expressed in this article are solely those of the authors and do not necessarily represent those of their affiliated organizations, or those of the publisher, the editors and the reviewers. Any product that may be evaluated in this article, or claim that may be made by its manufacturer, is not guaranteed or endorsed by the publisher.
