# Supplementary material for: Added survival benefit of whole brain radiotherapy in brain metastatic non-small cell lung cancer: Development and external validation of an individual prediction model
Source: Front Oncol. 2022 Nov 29;12:911835. doi: 10.3389/fonc.2022.911835 (PMC9796174; doi:10.3389/fonc.2022.911835)
Supplement: Supplementary file 1 [file DataSheet_1.docx]

**SUPPLEMENTARY**

Table S1 Survival prediction models for brain metastases

Table S2: Details of potential predictors

Table S3: Coefficients in Graded Prognostic Assessment for Lung Cancer Using Molecular Markers (Lung-mol GPA) index

Table S4: Univariate & multivariable Cox regression for overall survival

Table S5: Final individual risk prediction

Figure S1 Kaplan–Meier Curve for overall survival

Figure S2: Nomogram of the final model

Figure S3: Predicted median survival time from total points of the nomogram

**Table S1: Survival prediction models for brain metastases**


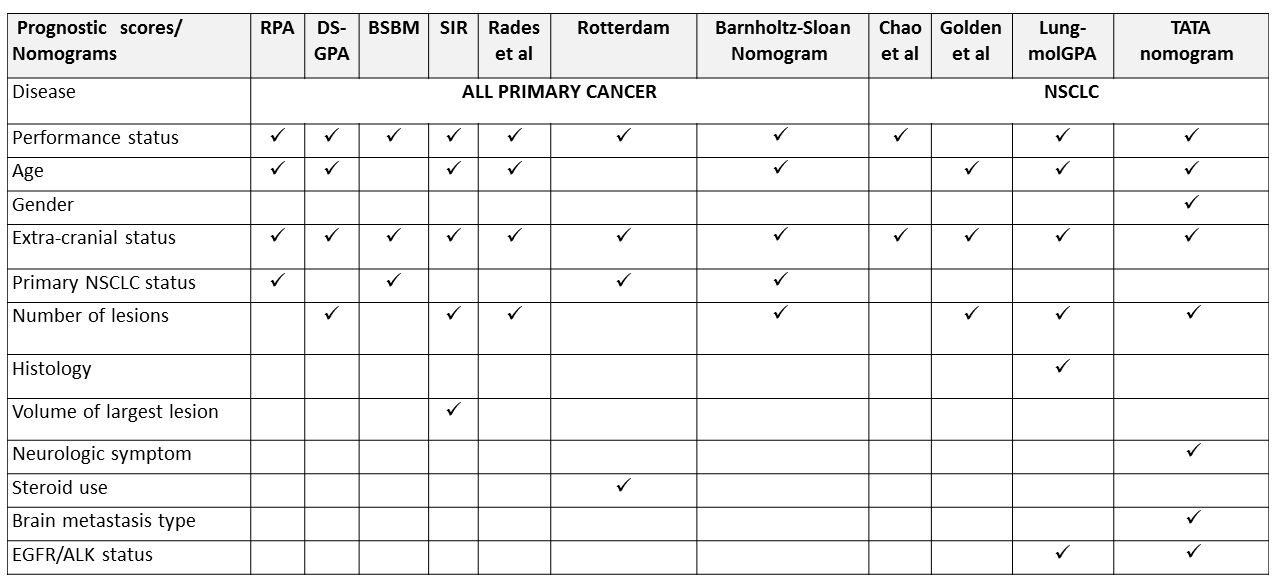


Abbreviations: ALK, anaplastic lymphoma kinase gene; BSBM, basic score for brain metastases^8^; DS-GPA, diagnosis-specific graded prognostic assessment^6-7^; EGFR, epidermal growth factor receptor; lung*-*molGPA, graded prognostic assessment for lung cancer using molecular markers^15^; NSCLC, non-small-cell lung cancer; RPA, recursive partitioning analysis score^4-5^; SIR, score index for radiosurgery^9^

**Table S2: Details of potential predictors**

| **Potential predictors** | **Scales and definitions** |
| --- | --- |
| Age at diagnosis (years) | Continuous variable |
| Gender | Binary variable: male/female |
| KPS score at diagnosis | Binary variable: < 70%/≥ 70% |
| Histology | Binary variable: adenocarcinoma/others |
| EGFR/ALK mutation | Polytomous variable: negative/positive/unknown |
| Neurological symptoms | Polytomous variable: none/mild *(any complained symptoms not fitting the major symptom criteria, for example, headache)*/major symptoms *(cranial nerve palsies, motor deficits, sensory deficits, cognitive deficits)* |
| Extracranial disease | Polytomous variable: controlled lung cancer and no extra-cranial metastasis/ controlled both lung cancer and extra-cranial metastasis/uncontrolled either lung cancer or extra-cranial metastasis/newly diagnosis lung cancer with any extra-cranial metastasis status (*evaluation within 3 months of the brain metastasis diagnosis)* |
| Previously received systemic treatment | Binary variable: yes/no |
| Measurable lesion | Binary variable: presence/absence *(measurable lesions defined by contrast-enhancing lesions at least one diameter size of 10 mm.)* |
| Received WBRT | Binary variable: yes/no |
| Received further systemic treatment | Binary variable: yes/no |

Abbreviations: ALK; anaplastic lymphoma kinase gene; EGFR; epidermal growth factor receptor; KPS, Karnofsky performance status; WBRT; whole brain radiotherapy

**Table S3: Coefficients in Graded Prognostic Assessment for Lung Cancer Using Molecular Markers (Lung-mol GPA) index**

|  | | **Adenocarcinoma** | | **Non-adenocarcinoma** | |
| --- | --- | --- | --- | --- | --- |
|  |  | **HR** | **coefficients** | **HR** | **coefficients** |
| Karnofsky performance status | 90–100 | 0.59 | -0.229 | 0.48 | -0.319 |
|  | 80 | 0.67 | -0.174 | 0.63 | -0.201 |
|  | 70 | 1 | 0 | 1 | 0 |
| Age | < 70 | 0.65 | -0.187 | 0.66 | -0.18 |
|  | 70 | 1 | 0 | 1 | 0 |
| Extra-cranial metastasis | Absent | 0.47 | -0.328 | 0.68 | -0.167 |
|  | Present | 1 | 0 | 1 | 0 |
| Number of brain metastases | 1–4 | 0.78 | -0.108 | 0.97 | -0.132 |
|  | > 4 | 1 | 0 | 1 | 0 |
| Genetic mutation | Positive | 0.48 | -0.319 | - | - |
|  | Negative/Unknown | 1 | 0 | - | - |

**Table S4: Univariate & multivariable Cox regression for overall survival**

| Characteristics | uHR  (95% CI) | *P* value | mHR  (95% CI) | *P* value |
| --- | --- | --- | --- | --- |
| Age | 1.01(1.00, 1.02) | 0.051 | 1.01(1.0, 1.02) | 0.137 |
| Female | 0.85(0.71, 1.03) | 0.096 | 0.88(0.68, 1.14) | 0.346 |
| KPS score > 70% | 0.53(0.44, 0.65) | < 0.001 | 0.79(0.59, 1.04) | 0.089 |
| Histology   - Adenocarcinoma - Non-adenocarcinoma | 1  1.12 (0.9, 1.4) | –  0.317 | 1  0.97 (0.71, 1.32) | –  0.852 |
| EGFR/ALK mutation   - Negative - Positive - Unknown | 1  0.59 (0.4, 0.87)  1.12 (0.84, 1.51) | –  0.008  0.424 | 1  0.54 (0.31, 0.92)  1.08 (0.72, 1.61) | –  0.025  0.725 |
| Neurological symptoms   - None - Mild - Major | 1  1.33 (0.95, 1.88)  1.82 (1.33, 2.48) | –  0.097  < 0.001 | 1  1.06 (0.67, 1.65)  1.38 (0.90, 2.10) | –  0.814  0.135 |
| Measurable lesions   - Absence - Presence | 1  0.90 (0.68, 1.21) | –  0.495 | 1  0.93 (0.64, 1.33) | –  0.681 |
| Extra-cranial disease  - Controlled lung and no ECM  - Controlled lung & ECM  - Uncontrolled lung or ECM  - First diagnosis lung with any ECM | 1  1.39 (0.88, 2.19)  1.89 (1.24, 2.89)  1.64 (1.13, 2.38) | –  0.153  0.003  0.009 | 1  2.56 (1.45, 4.51)  2.90 (1.75, 4.81)  4.0 (2.33, 6.87) | –  0.001  < 0.001  < 0.001 |
| Previous systemic treatment | 1.05 (0.87, 1.27) | 0.616 | 1.45 (1.0, 2.12) | 0.051 |
| Further systemic treatment | 0.34 (0.27, 0.42) | < 0.001 | 0.4 (0.28, 0.55) | < 0.001 |
| WBRT | 0.46 (0.37, 0.59) | < 0.001 | 0.50 (0.36, 0.70) | < 0.001 |

Abbreviations: ALK; anaplastic lymphoma kinase gene; CI, confidence interval; ECM, extra-cranial metastasis; EGFR; epidermal growth factor receptor; KPS, Karnofsky performance status; mHR, multivariable hazard ratios; uHR, univariable hazard ratios; WBRT, whole brain radiotherapy

**Table S5: Final individual risk prediction**

S (t) = S_(0)_t^exp(risk scores)^

S0(3^rd^ month)= 0.743

S0(6^th^ month)= 0.463

Risk scores = [(-0.160‬*female)

+(-0.317‬*KPS>70%)

+(0.160*Mild neurologic symptom)

+(0.305*Major neurologic symptom)

+(0.565*Controlled lung disease and ECM)

+ (0.816‬*Uncontrolled lung disease or ECM)

+(0.977*First diagnosed lung cancer with any ECM)

+(0.271*Previous systemic treatment)

+(-0.961‬*Received further systemic treatment)

+(-0.483‬*WBRT)]

**Figure S1** **Kaplan–Meier Curve for overall survival**


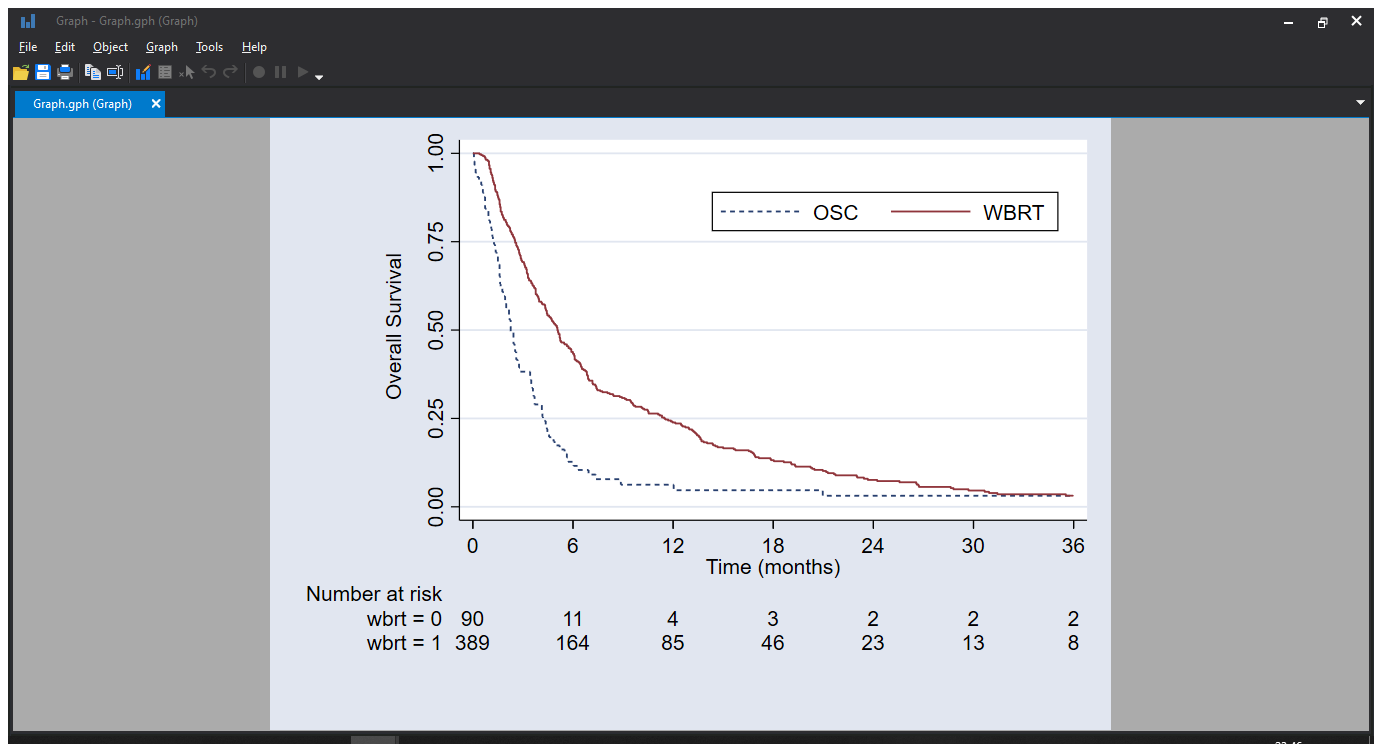


WBRT

OSC

Figure S1 Kaplan–Meier Curve for overall survival for patients received optimal supportive care (OSC) and whole brain radiotherapy (WBRT)


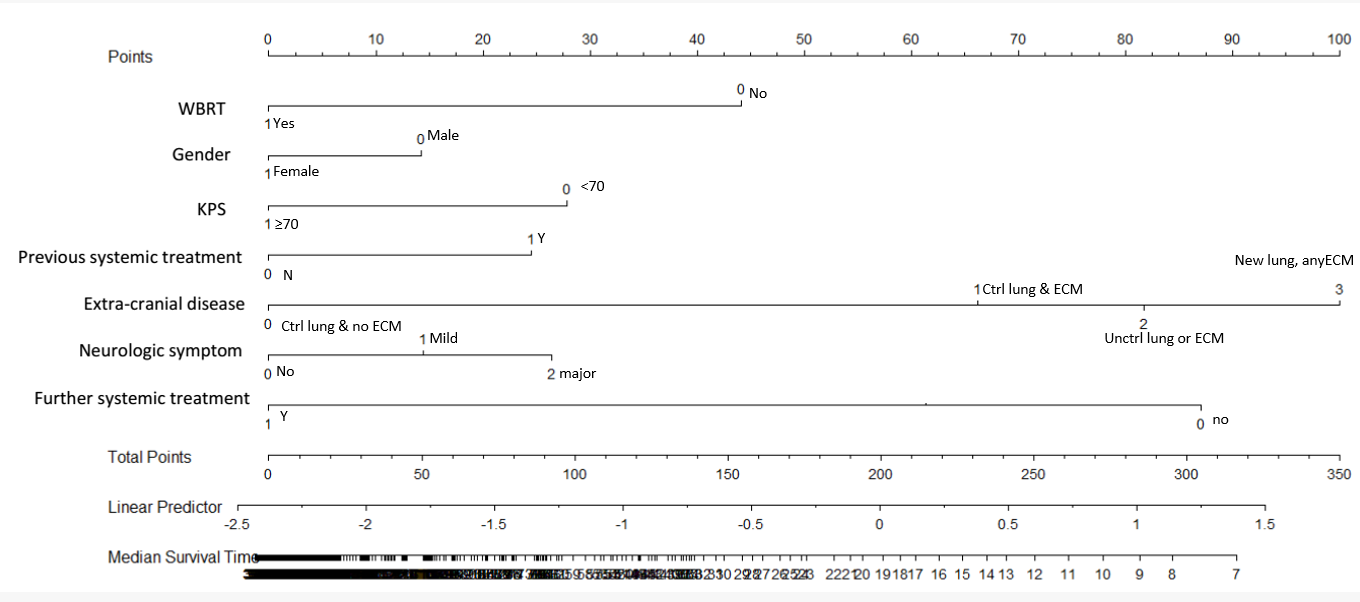


Please see predictors’ coding on the following page

**Figure S2: Nomogram of the final model**

| Predictors | Scales and definitions |
| --- | --- |
| Gender | 0 = male  1 = female |
| KPS | 0 = KPS < 70%  1 = KPS ≥ 70 % |
| Neurological symptoms | 0 = none  1 = mild (any complaint of symptoms that do not fit in the major symptom criteria, for example, headache)  2 = major symptoms (cranial nerve palsies; motor/sensory/cognitive deficits) |
| Extracranial disease | 0 = lung cancer controlled, but no extracranial metastasis  1 = both lung cancer and extracranial metastasis controlled  2 = neither lung cancer nor extracranial metastasis controlled  3 = newly diagnosed lung cancer with any extracranial metastasis status  (Evaluation performed within 3 months of the brain metastasis diagnosis) |
| Previously received systemic treatment | 0 = no  1 = yes |
| Received WBRT | 0 = no  1 = yes |
| Received further systemic treatment | 0 = no  1 = yes |

**Figure S3: Predicted median survival time from total points of the nomogram**

**Total Points**
